# Smoking treatments for patients with mental illness: case presentation and a brief literature review

**DOI:** 10.1192/j.eurpsy.2023.1593

**Published:** 2023-07-19

**Authors:** F. Garcia Sanchez, M. Gutierrez Rodriguez, C. Moreno Menguiano, M. A. Corral Alonso, J. J. Vazquez Vazquez, S. M. Bañon Gonzalez, V. Voces Domingo, J. A. Casado de la Hera

**Affiliations:** 1 Psychiatry, Hospital Universitario de Móstoles, Mostoles; 2 Psychiatry, Hospital Universitario Infanta Sofia, San Sebastian de los Reyes, Spain

## Abstract

**Introduction:**

Smoking prevalence in patients with mental illness ranges between two to 4 times higher than general population. This higher prevalence has a multifactorial origin, and some of the possible causes are still unknown.

They have a higher prevalence of tobacco-associated diseases and higher mortality.

Additionally, these patients have greater difficulty in treating and quitting smoking.

A relationship has been found between severity of mental illness and smoking. Risk of suicide seems to be higher in patients with higher tobacco consumption. Schizophrenia is the mental illness that has been most closely related to smoking, with a prevalence close to 90%.

**Objectives:**

The aim of this work is reviewing the current bibliography referring to smoking treatments for patients with mental illness

**Methods:**

A literature search using electronic manuscripts available in PubMed database published during the last ten years and further description and discussion of a single-patient clinical case

**Results:**

The treatment of tobacco dependence in patients with mental illnesses is sometimes waited until there is psychiatric stability, which can take a long time in those cases with more severe mental disorders, which can have negative physical and psychiatric consequences.

The combined treatment of cognitive behavioral therapy and pharmacological treatment is the most effective approach. Nicotine replacement therapy can be useful, while combined use of antidepressants or anxiolytics is also recommended.

Bupropion has shown efficacy. In patients with schizophrenia it does not seem to worsen positive symptomatology, but improving the negative one. It should not be used in patients with bipolar disorder or bulimia.

Varenicline has shown efficacy in the general population, but limitations were established in patients with mental illness, although it is the drug that has shown greater efficacy. However, is not currently available in our country.

Cytisine is a drug with limited number of studies in the psychiatric population but it may be a reasonable treatment alternative.

**Conclusions:**

The prevalence of tobacco use in patients with mental illness is higher than the general population, especially in paranoid schizophrenia. The consequences on physical health and the evolution of psychiatric illness are very relevant. Based on above, a multidisciplinary and coordinated management involving psychiatrists and other specialists in the treatment of these patients should be desirable.

**Disclosure of Interest:**

None Declared

